# Gut Permeability Might be Improved by Dietary Fiber in Individuals with Nonalcoholic Fatty Liver Disease (NAFLD) Undergoing Weight Reduction

**DOI:** 10.3390/nu10111793

**Published:** 2018-11-18

**Authors:** Marcin Krawczyk, Dominika Maciejewska, Karina Ryterska, Maja Czerwińka-Rogowska, Dominika Jamioł-Milc, Karolina Skonieczna-Żydecka, Piotr Milkiewicz, Joanna Raszeja-Wyszomirska, Ewa Stachowska

**Affiliations:** 1 Department of Medicine II, Saarland University Medical Center, Saarland University, 66421 Homburg, Germany; marcin.krawczyk@uks.eu; 2 Laboratory of Metabolic Liver Diseases, Centre for Preclinical Research, Department of General, Transplant and Liver Surgery, Medical University of Warsaw, 02-097 Warsaw, Poland; 3 Department of Biochemistry and Human Nutrition, Pomeranian Medical University, 71-210 Szczecin, Poland; domi.maciejka@wp.pl (D.M.); ryterska.karina@gmail.com (K.R.); majaczerwinska89@gmail.com (M.C.-R.); dominikajamiol@interia.pl (D.J.-M.); karzyd@pum.edu.pl (K.S.-Ż.); 4 Liver and Internal Medicine Unit, Department of General, Transplant and Liver Surgery of the Medical University of Warsaw, 02-097 Warsaw, Poland; p.milkiewicz@wp.pl (P.M.); jorasz@gmail.com (J.R.-W.); 5 Translational Medicine Group, Pomeranian Medical University, 71-210 Szczecin, Poland

**Keywords:** NAFLD, zonulin, fiber, diet

## Abstract

(1) Introduction: Zonulin (ZO) has been proposed as a marker of intestinal permeability. Only a few studies have analyzed to date how diet influences the serum concentration of ZO among patients with non-alcoholic fatty liver disease (NAFLD). We performed a six-month dietetic intervention to evaluate the association between fiber intake and ZO concentration in 32 individuals with NAFLD. (2) Methods: Fiber content in the diet was estimated by Food Frequency Questionnaire (FFQ) and by analyzing 72-h nutritional diaries. ZO concentrations in serum were measured before and after the intervention by immunoenzymatic assay (ELISA). Fatty liver was quantified using the Hamaguchi score before and after the dietetic intervention. (3) Results: During the intervention, the dietary fiber intake increased from 19 g/day to the 29 g/day concomitant with an increase in the frequency of fiber consumption. All patients experienced significant (all *p* < 0.05) improvements in serum aspartate aminotransferase (AST), alanine aminotransferase (ALT) and gamma-glutamyltransferase (GGTP) activities. We also detected decreased serum triglycerides (*p* = 0.036), homeostatic model assessment insulin resistance (HOMA-IR (*p* = 0.041) and insulin content (*p* = 0.34), and improvement of fatty liver status according to the Hamaguchi score (*p* = 0.009). ZO concentration in serum decreased by nearly 90% (7.335 ± 13.492 vs. 0.507 ± 0.762 ng/mL, *p* = 0.001) and correlated with the amount of dietary fiber intake (*p* = 0.043) as well as the degree of fatty liver (*p* = 0.037). (4) Conclusion: Increasing nutritional fiber results in reduced serum ZO levels, reduced liver enzymes and improved hepatic steatosis in patients with NAFLD, possibly by altering intestinal permeability. Increased dietary fiber intake should be recommended in patients with NAFLD.

## 1. Introduction

The gut epithelium represents a key protective barrier separating internal organs from the adverse environment of the gut lumen [1]. Its permeability is controlled by several multiprotein adhesive complexes including tight junctions (TJ), subjacent adherens junctions (AJ), and desmosomes [1]. The intestinal barrier is a highly dynamic structure shaped by interactions with internal and external stimuli, such as cytokines, growth factors, and bacteria [1,2,3]. Diet-derived substances also interact with the barrier and modulate its permeability. For instance, glutamine [4,5] and tryptophan [6], peptides derived from cheese and milk [7,8], and polyphenols [9] act as positive regulators of epithelial permeability, whereas other compounds such as gliadin [10] or medium-chain fatty acids [11,12] negatively regulate intestinal barrier permeability. Zonulin (ZO) is a 47-kDa protein that regulates intestinal permeability by modulating intracellular TJ structure and function [13]. The secretion of ZO into serum is believed to be regulated by several factors, including components of the diet [14,15].

Nonalcoholic fatty liver disease (NAFLD) is now the most common cause of chronic liver disease. It is currently detected in over 20% of Europeans, and its prevalence is predicted to increase over the years [16,17]. NAFLD is considered the hepatic manifestation of the metabolic syndrome, and the steatotic phenotype appears to be worsened by disruptions in the host gut microbiota [18,19]. An increasing body of evidence demonstrates that NAFLD correlates positively with the phenomenon of “leaky gut”, the mechanism encompassing TJ damage [20] in both young patients [13] and adults [21,22]. The disturbances of the gut-liver axis might explain, at least to a certain extent, the phenotype of fatty liver [23]. For example, gut dysbiosis and skewed production of inflammatory markers, among others lipopolysachcaride, might modulate the progression of NAFLD. This is underscored by the association between endotoxemia and severe forms of fatty liver [24]. Moreover, a dysbiotic gut ecosystem might result in increased production of endogenous ethanol, which could disrupt the integrity of the intestinal barrier and further increase liver injury in the setting of increased hepatic fat accumulation [25]. Several studies have reported that ZO concentrations increase significantly with the severity of steatosis [13,20,21] and positively correlate with body mass index (BMI), liver histopathology, fasting insulin, and HOMA-IR as well as concentrations of inflammatory markers (e.g., serum IL-6) [21]. To the best of our knowledge, there is only one study that has described how a reduction diet among NAFLD patients might influence the serum concentration of ZO [13]. Here we present the impact of a six-month dietetic intervention, comprising an increased intake of fiber and a decreased intake of fat and simple sugars, on the liver status and ZO serum concentrations in a cohort of 32 patients with NAFLD.

## 2. Materials and Methods

### 2.1. Study Design

**Figure 1:**
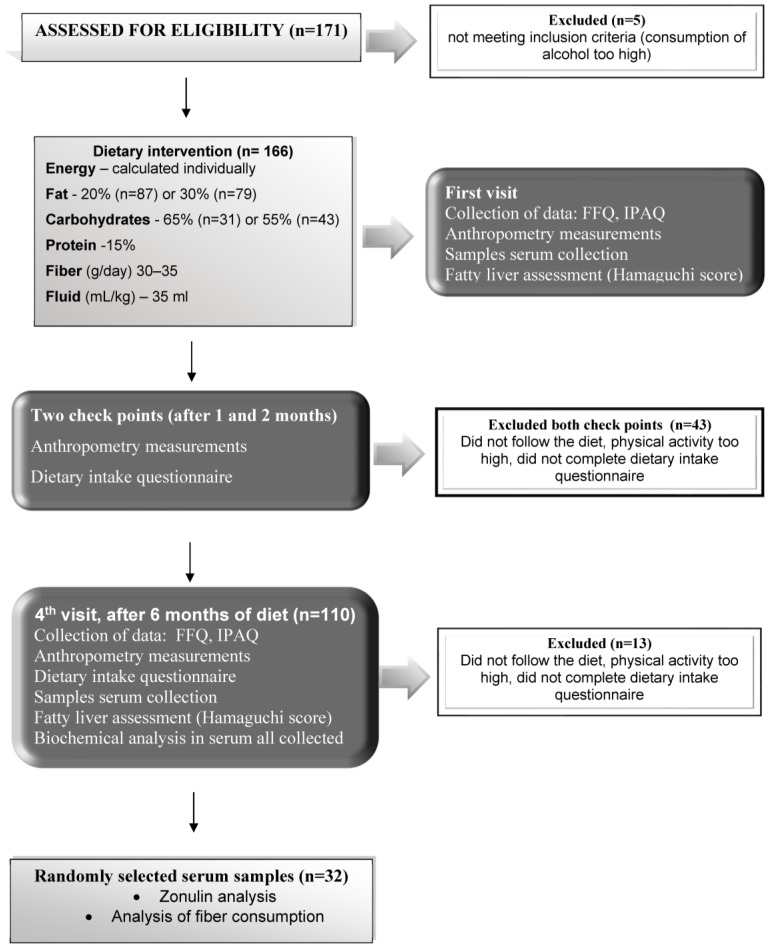
Flowchart for the inclusion of individuals in the study.

In the present study we included subjects selected from the Nutrient-Induced Insulin Output Ratio (NIOR) study, which was intended to investigate nutritional strategies for the individualized treatment of NAFLD [22]. In brief, this study was designed as a randomized controlled intervention trial to compare the influence of three nutrigenetic dietary strategies on fatty liver during a six-month intervention [26]. The exclusion criteria were: Diabetes mellitus (DMII); chronic and acute liver diseases other than NAFLD; high levels of physical activity (>3000 kcal/week in leisure-time physical activity); changes in physical activity during the dietary intervention; use of statins; any condition that could limit the mobility of the participant; not being able to attend control visits; vegetarianism or a need for other special diets; excessive consumption of alcohol (more than 20 g in women and more than 30 g in men, per day); and other drug addiction. Compliance with the diet was evaluated at 3 control points (at 1, 2, and 6 months after commencement of the diet) (Figure 1). All subjects were instructed to maintain their usual physical activity (metabolic equivalent of the task; MET), which was controlled by a 2-h physical activity questionnaire. Initially, 171 Caucasian adults were enrolled in the NIOR study; however, 5 subjects did not meet the inclusion criteria. After the first month of the study further 29 individuals were excluded from the study: Either they did not comply with the prescribed diet, engaged in too much physical activity or they failed to complete the dietary intake questionnaire.

At the second control meeting (i.e., 2 months after the beginning of the diet), 14 participants were excluded from the study for similar reasons. During the final visit (after 6 months), a further 13 participants were excluded. As a result, a total of 110 participants were adherent to the intervention protocol and remained in the study (Figure 1). We used an online randomization tool [26] to select patients for biochemical analyses. Serum samples from 32 participants (22 males and 10 females) were available for the current analysis. The fiber consumption was analyzed (soluble fiber as well as insoluble fiber) with the help of the Fineli base (https://fineli.fi/fineli/en/index) and the Diet program (IZZ Poland). The study protocol was approved by the ethics committee of the Pomeranian Medical University and conformed to the ethical guidelines of the 1975 Declaration of Helsinki (Szczecin, Poland, 25 01 2010 KB-0012/09/10). Participants provided written informed consent before the study.

### 2.2. The Nutritional Intervention and Control

The NIOR diet was modified on the basis of the of the American Heart Association (AHA) guidelines, including 3–5 meals/day, modified by the genetic result of NIOR as described in detail [27]. Menus were prepared as a daily plan for the 7 days of the week and included guidance on the daily timing of the five meals.

Nutrition patterns were analyzed with a Food Frequency Questionnaire (FFQ) and a 72-h food diary (including 2 working days and one day free of work) [27]. Fiber content (soluble and insoluble) was estimated by means of two methods: frequency of consumption by means of the FFQ and by analyzing the 72 nutritional diaries (from the three control points) via the Diet 5 program (IZZ Poland) and Fineli (the Finnish database). Dietary content, nutritional sources and the products used are presented in Table 1. Three portions of vegetables and two portions of fruit were introduced to the patients’ diet. The recommended sources of insoluble fiber were as follows: Whole wheat bread, whole-wheat pasta, cereal and brown rice, whole rye bread, and graham bread. Recommended vegetables were: Tomatoes, savoy cabbages (fresh and pickled), napa cabbages, green beans, paprika, fresh and pickled cucumbers, onions, chives, red beans, lentils, spinach, carrots, leeks, celery, broccoli, lettuce, and dill. Recommended fruits were: apples, plums, and apricots (including dried). Finally, recommended seeds and nuts were: pumpkin seeds and walnuts. The level of physical activity was assessed using the questionnaire technique with validated International Physical Activity Questionnaire (IPAQ). Physical exercise was estimated with MET units (min/week). During the first and final visit, the degree of fatty liver was evaluated by a trained physician according to the Hamaguchi score [28], using a high-resolution B-mode abdominal ultrasound scanner (Acuson X300). Hamaguchi score ≥ 2.0 was set as the cut-off for NAFLD [28].

### 2.3. Laboratory Analyses

Venous fasted blood samples were collected into tubes containing anticoagulant Ethylenediaminetetraacetic acid (EDTA). Blood samples were centrifuged at 3500 rpm for 10 min at 4 °C within 2 h of collection. Standard blood biochemical analyses were carried out at the University Hospital Laboratory (Szczecin, Poland). Plasma ZO concentrations were measured by ELISA (Immundiagnostik AG, Bensheim, Germany) according to manufacturer’s instructions.

### 2.4. Statistics 

For statistical analyses, R software 3.0.2 was used and all results were expressed as mean and standard deviation. Since the distribution in most cases deviated from normality (Shapiro-Wilk test), non-parametric paired tests were used (Wilcoxon signed-rank test) and *p* < 0.05 was considered as statistically significant. The correlations between quantitative variables were calculated using the Spearman’s correlation test.

## 3. Results

### 3.1. The Amount of Zonulin in Serum Decreases with the Improvement of Fatty Liver

**Figure 2:**
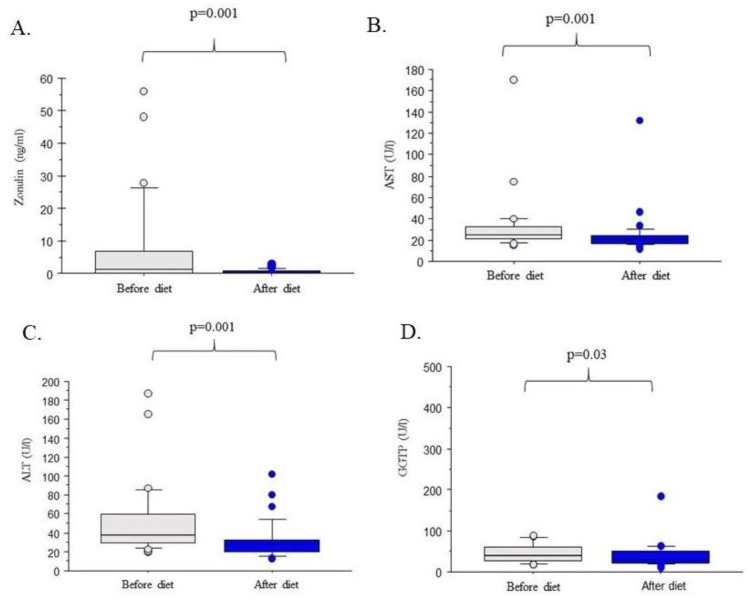
Zonulin concentration and liver function tests before and after the dietetic intervention. (**A**) Zonulin concentration. (**B**) Aspartate aminotransferase (AST) activity. (**C**) Alanine aminotransferase (ALT) activity. (**D**) Gamma-glutamyltransferase (GGTP) activity.

As shown in Figure 2, the six-month dietetic intervention resulted in a significant improvement of liver injury markers in the studied group of 32 individuals as reflected by decreases of serum AST (*p* = 0.001), ALT (*p* = 0.001) and GGTP (*p* = 0.03). As shown in Table 2, it also led to a reduction of the Hamaguchi score (2.87 ± 0.59 vs. 1.40 ± 0.93; *p* = 0.009). Furthermore, serum triglyceride concentrations (*p* = 0.036), HOMA-IR (*p* = 0.041) index as well serum insulin (*p* = 0.34) decreased as a result of the dietetic intervention. Notably, we also detected a significant (*p* = 0.001), almost 90% reduction of serum ZO concentration as shown in Figure 2A. Table 3 demonstrates that serum ZO correlated with the degree of fatty liver (i.e., with Hamaguchi score (*p* = 0.037) serum AST (*p* = 0.041) and ALT (*p* = 0.043)), total lipids (*p* = 0.041), as well as low-density lipoprotein (*p* = 0.029). Interestingly, we detected a negative correlation between high-density lipoprotein and serum ZO contents (Table 3) but no significant link between ZO concentration and BMI or glucose metabolism parameters (all *p* > 0.05).

### 3.2. The Content of Zonulin in the Serum May be Related to the Increase in Fiber Consumption

The analysis of nutritional diaries showed that all patients included in the analysis followed the suggested nutritional recommendations. No significant deviations from the proposed energy supply or key nutrients intake (proteins, carbohydrates and fats) were observed. Recruited patients consumed on average 29.24 ± 10.97 g fiber per day, which is higher than the fiber intake as estimated for the Polish population i.e., 19 g per day [29]. This included: soluble fiber (6.69 ± 3.21 g per day) and insoluble fiber (17.44 ± 7.11 g per day). These calculations were done based on a 72-h recall diary subjected to the Fineli base. According to the database of the Institute of Nutrition in Poland (IZZ), the calculated fiber content was slightly lower: 28.18 g per day. We estimated that the frequency of consumption of fruits and vegetables rose among study participants from a few portions per week to a few portions per day (Table 4). The Spearman’s rank analysis indicated the existence of a correlation between the amount of fiber in the diet and the concentration of ZO in blood (*p* = 0.043) (Table 5). No other analyzed food constituent proved to correlate with serum ZO (*p* > 0.05).

## 4. Discussion

NAFLD is turning into the most frequent liver condition worldwide, and diet is regarded as one of the major drivers of the steatotic phenotype. In our study we reconstructed the amount and frequency of dietary fiber consumption in patients with NAFLD and detected a correlation between ZO concentrations and fiber intake. Previously Pacifico et al. [13] demonstrated that serum ZO is elevated in the blood of obese children with NAFLD: ZO values correlated with steatosis, but not with fibrosis score, or the presence of nonalcoholic steatohepatitis (NASH). The results presented in the study by Hendy et al. [21] suggest that ZO levels could be used for a quick and noninvasive diagnosis of liver inflammation. It was observed that once ZO serum concentration in a patient with NAFLD exceeds 8.3 ng/mL, the risk of developing NASH rises considerably. This might be significant for the development of non-invasive biomarker panels that could substitute liver biopsy [21].

In our study, we examined whether ZO concentrations decreased during the introduction of calorie restriction. Damms-Machado et al. [30] evaluated ZO contents in the stool of patients who underwent a 12-month dietary intervention and showed that ZO concentrations in stool are not influenced by weight reduction therapy and do not correlate with anthropometric variables. In our study we did not detect any significant correlations between serum ZO concentrations and the reduction of body mass parameters either. Similarly to Damms-Machado et al. [30], we observed a correlation between ZO and lipid parameters, enzymatic markers of liver injury and the degree of steatosis. Damms-Machado et al. observed that ZO concentrations among patients did not change significantly with steatosis over the course of the study [30]. They concluded that the lack of fecal ZO alterations after the intervention was a result of ZO-independent regulation of TJ occurring during the course of weight reduction, which leads to the differences in gut permeability. We previously described a similar phenomenon (ZO-independent regulation) in a study in which we analyzed the permeability of the barrier after the use of colostrum [15].

We speculate that changes in fiber intake were responsible for the reduction of ZO in the blood of our patients. Indeed, the level of dietary fiber rose in the diet of our patients regardless of the supply of other nutrients (i.e., fat or carbohydrates). Our patients were subjected to control visits four times during the 6-month dietary intervention, and only those patients who significantly increased their frequency of consumption and supply of soluble and insoluble fiber completed the study. A recently published study found that fruit fiber consumption was associated with improved liver health [31]. According to these results it was postulated that higher insoluble fiber consumption (≥7.5 g/day) leads to improvements in fatty liver parameters. Notably, a regression model proved a relationship between liver status and fruit derived fiber [31]. Our study has some limitations that should be kept in mind when interpreting the data. First of all, included patients were not biopsied, hence we did not have data on the presence of NASH. For the same reason, we did not have data on the degree of liver fibrosis in our patients. We focused on the measurements of zonulin, however other markers of intestinal permeability such as bacterial endotoxin levels, LBP protein concentration or serum citrulin might also provide additional insights into observed changes during diet. Finally, the studied cohort was relatively small and the Hamaguchi score [28], which we applied to quantify NAFLD, has not been thoroughly validated in Caucasians.

Why might the above results be of significance? Fibers fermenting in the intestine provide short-chain fatty acids that fulfill a protective and nourishing role for colonocytes, ensuring the preservation of the intestinal barrier [32]. Plant fiber provides nourishment for intestinal microbiota, which are of prime importance to the preservation of the intestinal barrier integrity [31]. The results of our study imply that appropriate fiber intake helps to maintain the proper structure and function of the intestinal barrier. The lifestyle recommendations for successful management of NAFLD promote fruit and vegetables in the diet of NAFLD patients. As our results show, the increased supply of fiber positively influences the NAFLD-associated parameters and it should be promoted among the specialists designing the diets for patients with fatty liver.

## Figures and Tables

**Table 1:** The nutritional products used in the diet.

Variable	Recommendation	Recommended Sources
Energy	Calculated Individually	
Fiber (g/day)	30–35	3 portions of vegetables a day; 2 portions of fruit as: fresh, fermented or boiled, wheat bread, whole-wheat pasta, cereal and brown rice
Fat as percentage of total calories (%)	20–30	Vegetable fats with a predominance of rapeseed oil and olive oil.
Carbohydrates (low and medium IG), percentage of total calories (%)	55–65	Low and medium glycemic index as: wheat and mix rye/wheat bread, whole-pasta, cereal and brown rice, fruit and vegetables.
Simple carbohydrate, percentage of total calories (%)	5–10	Dry fruits
Protein (%)	15	Poultry (chicken and turkey), fish (oily fish 3 times a week), fermented dairy products (2 times a day), eggs (4–5 a week), lean cottage cheese, cheese with reduced fat content.
Fluid (mL/kg)	35	Water, coffee (1–2 cup a day) tea (black, green).
Vitamins and minerals	Consistent with recommended daily allowance	Natural sources from vegetables and fruit

**Table 2:** Baseline characteristics of the study cohort (*n* = 32), as well as effects of the dietetic intervention.

Parameters	Before Diet	After Diet	*p* Value
Age (year)	48.03 ±13.13	-	-
Body Mass (kg)	98.76 ± 19.80	91.64 ± 16.60	NS
BMI (kg/m^2^)	33.19 ±5.71	30.91 ± 5.45	NS
Fat mass (kg)	38.08 ± 6.06	32.84 ± 9.41	NS
Lean body mass (kg)	60.93 ± 12.90	58.48 ± 12.04	NS
Water content (kg)	45.25 ± 9.16	43.81 ± 8.07	NS
Fatty liver (Hamaguchi score)	2.87 ± 0.60	1.40 ± 0.93	0.009
Triacylglycerols (mg/dL)	219.09 ± 326.01	166.31 ± 219.71	0.036
Total cholesterol (mg/dL)	206.06 ± 52.54	200.56 ± 45.44	NS
High density lipoprotein (mg/dL)	44.84 ± 10.09	49.47 ± 14.47	NS
Low density lipoprotein (mg/dL)	121.15 ± 33.43	122. ± 60.32	NS
Total lipids (mg/dL)	731.06 ± 414.63	706.44 ± 300.43	NS
Glucose (mg/mL)	104.50 ± 20.01	100.78 ± 10.72	NS
Insulin (U/mL)	17.90 ± 11.44	7.47 ± 5.34	0.034
HOMA-IR	4.85 ± 3.14	1.89 ± 1.36	0.041

Abbreviation: BMI, body mass index; HOMA-IR, homeostasis model assessment of insulin resistance; NS, not significant.

**Table 3:** Correlations between serum zonulin content and patient characteristics.

Parameters	RHO	*p* Value
Age (years)	0.05	NS
Body Mass (kg)	<0.01	NS
BMI (kg/m²)	(−) 0.18	NS
Fat mass (kg)	(−) 0.13	NS
Fat content (%)	(−) 0.17	NS
Lean body mass (kg)	0.09	NS
Water content (kg)	0.13	NS
Aspartate transaminase (U/L)	0.35	0.041
Alanine transaminase (U/L)	0.36	0.043
Gamma-glutamyltransferase (U/L)	0.19	NS
Triacylglycerols (mg/dL)	0.17	NS
Total cholesterol (mg/dL)	0.22	NS
High density lipoprotein (mg/dL)	(−) 0.30	0.029
Low density lipoprotein (mg/dL)	0.37	0.032
Total Lipids (mg/dL)	0.31	0.041
Glucose (mg/mL)	(−) 0.07	NS
Insulin (U/mL)	(−) 0.10	NS
Fatty liver (Hamaguchi score)	0.33	0.037

**Table 4:** Changes in patients dietary patterns during the intervention.

Frequency of Consumption According FFQ	Before Intervention	During the Diet	Change in Frequency
Dairy products	Every day	Every day	No change
Cereal products	A few times a day	A few times a day	No change
Fats	Every day	Every day	No change
Fruits	A few times a week	A few times a day	Increased
Vegetables and grains	A few times a week	A few times a day	Increased
Poultry, meet and fish	Every day	Every day	No change

Abbreviation: FFQ, food frequency questionnaire.

**Table 5:** Correlations between diet compounds and the concentration of serum zonulin.

Parameters	RHO	*p* Value
Energy of diet (kcal)	0.01	NS
Protein (%)	(−) 0.01	NS
Fat (%)	(−) 0.07	NS
Saturated fatty acids (%)	(−) 0.02	NS
Monounsaturated fatty acids (%)	(−) 0.03	NS
Polyunsaturated fatty acids (%)	(−) 0.20	NS
Carbohydrates (%)	0.07	NS
Fiber (%)	(−) 0.30	0.043
